# Cortical activation during imagined walking for people with lower limb loss: a pilot study

**DOI:** 10.3389/fnhum.2023.1163526

**Published:** 2023-07-05

**Authors:** Susan W. Hunter, Aysha Motala, Alicia E. Cronin, Robert Bartha, Ricardo Viana, Michael W. Payne

**Affiliations:** ^1^School of Physical Therapy, The University of Western Ontario, London, ON, Canada; ^2^School of Psychology, The University of Stirling, Stirling, Scotland; ^3^Department of Medical Biophysics, Schulich School of Medicine and Dentistry, The University of Western Ontario, London, ON, Canada; ^4^Centre for Functional and Metabolic Mapping, Robarts Research Institute, The University of Western Ontario, London, ON, Canada; ^5^Department of Physical Medicine and Rehabilitation, Schulich School of Medicine and Dentistry, The University of Western Ontario, London, ON, Canada

**Keywords:** limb amputation, functional MRI, motor cortex, mental imagery, walking

## Abstract

Each year in Canada, a substantial number of adults undergo limb amputation, with lower limb amputation (LLA) the most prevalent. Enhancing walking ability is crucial for optimizing rehabilitation outcomes, promoting participation, and facilitating community reintegration. Overcoming challenges during the acute post-amputation phase and sub-acute rehabilitation necessitates alternative approaches, such as motor imagery and mental practice, to maximize rehabilitation success. However, the current evidence on activation patterns using motor imagery in individuals with LLA is limited. The primary objective was to assess the feasibility of observing brain activation during imagined walking in individuals with LLA utilizing 3T functional magnetic resonance imaging (fMRI). Eight individuals with LLA and 11 control subjects participated. Consistent with representations of the lower limbs, both control and amputee groups demonstrated bilateral activation in the medial surface of the primary motor and somatosensory cortices. However, individuals with lower limb amputations exhibited significantly greater activation during imagined walking, particularly in frontal regions and the medial surface of the primary motor and supplementary motor cortices. Furthermore, the volume of activation in the bilateral primary motor cortices was higher for participants with amputations compared to controls. The protocol developed in this study establishes a foundation for evaluating the effects of a gait training program that incorporates mental imagery alongside conventional rehabilitation practices, in contrast to standard care alone. This pilot investigation holds potential to enhance our understanding of brain plasticity in individuals with LLA and pave the way for more effective rehabilitation strategies to optimize functional recovery and community reintegration.

## Introduction

Each year in Canada approximately 7,400 people over the age of 18 years have a limb amputation, with lower limb amputation (LLA) being the most common (Imam et al., 2017). Rehabilitation is essential after limb loss for restoring independent mobility and enhancing quality of life. Successful mobility has been identified by people with LLA as the single most important contributor to quality of life after limb loss (Asano et al., 2008). Falls are the greatest threat to mobility and are very common with 52% of people with LLA sustaining at least one fall each year. Most falls occur while people are using their prosthesis during walking (Kulkarni et al., 1996). Improving walking will prevent falls and increase participation and quality of life (Hunter et al., 2017).

Even after intensive rehabilitation to use a prosthesis, walking problems are common (Miller et al., 2001). Developing new interventions to improve walking ability will optimize rehabilitation for people with LLA to achieve their potential for community reintegration and participation. Given that the timing of commencing prosthetic rehabilitation may be limited in the acute post-amputation period due to post-operative pain and wound healing, and in sub-acute rehabilitation due to limited endurance initially for use of the prosthesis, alternative approaches such as motor imagery and mental practice added to routine rehabilitation practice may be appropriate to optimize ability. Motor imagery involves asking individuals to picture themselves performing specific motor activities without actual execution of the movement (Jeannerod, 1994). Motor imagery training of walking has been successfully used as a rehabilitation tool for gait retraining among people with stroke (Heremans et al., 2011; Verma et al., 2011). Importantly, imagined walking has been found to activate the same areas of the brain as the actual process of walking (Cremers et al., 2012; Blumen et al., 2014).

Success of a motor imagery protocol among people with LLA requires being able to demonstrate that brain activation can be elicited. This may be limited in people with LLA due to functional reorganization of the brain after the amputation that can result in altered activation patterns for the lower extremity (Florence and Kaas, 1995; Chen et al., 2002). Motor imagery ability is also known to vary across individuals regardless of amputation status, influencing the vividness of the images for both visual and kinesthetic components of imagery (Van der Meulen et al., 2014). This variation in ability can impact cerebral activation during imagined tasks, such as walking, specifically good imagers demonstrate better recruitment of motor areas in imagined walking (Van der Meulen et al., 2014). Mental representation of motor activities can be enhanced with practice, though the ability to generate vivid images can be weakened with limb loss (Malouin et al., 2009). The Graded Motor Imagery program which involves progressive tasks of left/right judgment, imagined movements and mirror therapy has been advocated for use in people with LLA as a focus for the reduction of phantom limb pain (Limakatso et al., 2020). The use of motor imagery for gait re-education in people with LLA has limited focus in the literature, meriting further work to link motor imagery to recovery of functional motor tasks.

An understanding of the activation patterns using motor imagery for people with LLA is limited Cruz et al. (2003) performed a descriptive analysis of motor cortex activation in three people with LLA. While active knee flexion and extension resulted in motor cortex activation, the study was not able to elicit any cortical activation with imagined knee flexion and extension (Cruz et al., 2003). A successful motor imagery protocol for people with LLA should elicit brain activation and this has not been demonstrated for people with LLA during imagined walking. Research is needed to better understand the brain activation during motor imagery and mirror therapy, disentangling motor execution of the phantom limb from motor imagery with the phantom limb, motor imagery that does not involve movement of a phantom limb to facilitate of optimal rehabilitation protocols and knowledge translation into clinical practice (Raffin et al., 2012; Barbin et al., 2016; Guerra et al., 2017).

Functional magnetic resonance imaging (fMRI) is the optimal method to evaluate task-based brain activation patterns. The primary objective of the study was to examine the feasibility of observing brain activation during imagined walking in people with lower limb loss. Secondary objectives were: (1) to compare the global and primary motor cortices activation during imagined walking between controls and people with unilateral transtibial amputation, and (2) to explore if activation is associated with motor imaging ability (MIQ-R).

## Methods

### Study design and participants

This study was a cross-sectional design using a sample of people with unilateral trans-tibial LLA and healthy controls. People with LLA were recruited from the outpatient Amputee Rehabilitation Program at Parkwood Institute, London, Canada. Control subjects were recruited from the community through e-newsletters. The MRI took place at the Centre for Functional and Metabolic Mapping, Robarts Research Institute at The University of Western Ontario, London, Canada. Ethics approval was obtained from The University of Western Ontario Health Science Research Ethics Board (HSREB#115626). Participants provided written informed consent to participate in the study.

The inclusion criteria for the control participants were right-hand dominant adult males (≥18 years) who were able to ambulate independently for 50 m with or without a gait aid. The inclusion criteria for the people with LLA were right-hand dominant adult males (≥18 years), unilateral trans-tibial amputation (right or left sided amputation), able to ambulate independently for 50 m using a prosthesis with or without a gait aid, wears a prosthesis daily (≥8 h), phantom limb pain was not a prevalent complaint [defined as occurring less than daily and measured ≤3/10 on a visual analog scale (0 = no pain, 10 = worst possible pain)] and medication usage was stable for at least 3 months. The exclusion criteria for all participants were non-English speaking, cognitive impairment that prevented providing informed consent, amputation of another body part, presence of an active psychiatric disorder, progressive neurological disease, previous neurosurgery/neurotrauma, previous total knee arthroplasty, or contraindications for undergoing MRI. The study required participants to attend two visits that occurred within a 2-week period. The first visit consisted of the collection of demographic and clinical information, and measurement of motor imagery ability. A training session was provided at the end of the first session to familiarize participants with the testing protocol to be used in the second visit for the fMRI scanning. The second visit consisted of the fMRI data collection. Data were collected between August 2021 and July 2022.

### Outcome measures

#### Sociodemographic and clinical characteristics

The following demographic and clinical information were collected for both groups: age (years), number of prescription medications, gait aid use, fear of falling, self-report rating of quality of health (5-point Likert scale ranging from poor to excellent), and employment status. A fall was defined as an unexpected event in which the participant comes to rest on the ground, floor, or lower level (Lamb et al., 2005).

The following information was collected for the people with LLA: etiology of amputation, duration of prosthesis use (years), prosthesis use (days per week), Socket Comfort Score (Hanspal et al., 2003) [visual analog scale with anchors of 0 (most uncomfortable) and 10 (most comfortable)] and Prosthetic Limb Users Survey of Mobility (PLUS-M) – 12 item short form (Haffner et al., 2017). The PLUS-M evaluates the perceived ability of people with LLA to carry out 12 activities that require use of both lower limbs while using their prosthesis (Haffner et al., 2017). Scores can range from 12 to 60 with higher scores representing better perceived mobility. Information on the presence of pain, open wounds, swelling, loss of sensation, phantom limb pain, hypersensitivity, and contractures related to the stump and intact limb was collected.

#### Motor imagery ability

The Revised – Movement Imagery Questionnaire (MIQ-R) assesses visual and kinesthetic movement imagery abilities (Gregg et al., 2010). The 14 item self-report MIQ-R is comprised of two subscales: visual imagery (7 items) and kinesthetic imagery (7 items). The imagined motor tasks involve the upper limb (5 items), lower limb (1 items), and trunk (1 item), which are repeated twice to evaluate visual and kinesthetic ability separately. Participants assumed a starting position unique to each movement and actively completed each described movement (no score is assigned to the active task). Following the active movement, participants were asked to imagine the movement just performed without physically executing the movement. Upon completion of the imagined task, the participant was asked to rate their ability to imagine the motor task on a 7-point scale (1 = very hard, 7 = very easy) for visual clarity and kinesthetic intensity. Scores were summed to determine an overall imagery score and sub-scores for visual imagery and kinesthetic imagery. Total scores can range from 14 to 98 and each subscale score can range from 7 to 49, higher scores indicate higher motor imagery ability.

#### Imaging protocol

Imaging was conducted on a 3T Siemens Prisma Fit MRI scanner using a 32-channel head coil to acquire all data. Each scan included the acquisition of a 3D volumetric T_1_-weighted sagittal inversion-prepared magnetization prepared rapid acquisition gradient echo (MP-RAGE) sequence (matrix size 256 × 256, 176 axial slices, 1 mm isotropic, repetition time (TR)/echo time (TE)/inversion time (TI) = 2300/2.98/900 ms, flip angle = 9°) covering the entire brain to produce images with high gray matter/white matter contrast. Blood oxygen level-dependent (BOLD) images were acquired using an interleaved echo-planar imaging sequence (matrix size 84 × 84, 48 axial slices per volume, 2.5 mm isotropic, TR/TE = 1000/30 ms, flip angle = 40°, iPAT = 2). The total BOLD acquisition time was 5 min and 30 s. For each condition, 30 volumes (30 s) were acquired for six (resting condition) and five (task condition) blocks, totaling 330 volumes per acquisition of 5 min and 30 s.

### Study design

A block paradigm task, which included 11 segments (six resting and five active), was performed to activate the motor pathway. Participants were instructed to close their eyes and visualize themselves walking (imagined walking) down a corridor at their usual comfortable pace. Participants imagined performing this task for 30 s, which was initiated and ended with an auditory cue for each of the five active blocks. Auditory cues were created using an in-house program developed in MATLAB (Mathworks, Natwick, MA, USA) v. R2020a and Psychtoolbox v.3.0.15. One fMRI run was completed for each subject.

### Imaging processing

Anatomical and functional images were preprocessed using the fMRI pipeline (*fmriprep*) version 1.5.4 (Esteban et al., 2019, 2022). Anatomical images were corrected for intensity non-uniformity, skull stripped, and spatially normalized. Brain tissue segmentation of cerebrospinal fluid (CSF), white matter (WM), and gray matter (GM) were performed on the skull-stripped brain, and brain surfaces were reconstructed using FreeSurfer v.6.0.1. Functional images underwent 3D motion correction and slice timing corrections. Co-registration between the anatomical T_1_-weighted image and functional images was executed using boundary-based registration (BBR) with 6 degrees of freedom using FreeSurfer. The anatomical and functional data were all converted and reported in MNI space. For further details of the *fmriprep* pipeline, please refer to the online documentation: https://fmriprep.org/en/latest/workflows.html.

A general linear model was run separately for each subject to assess brain activity related to the proposed block design. First, spatial smoothing was conducted using a 6 mm full-width-half maximum Gaussian kernel in FSL v.6.0. The predictors of each subject were modeled by convolving the block paradigm boxcar function with a double-gamma hemodynamic response function. The nuisance regressors were motion-related parameters, which consisted of three regressors for each translation and rotation direction.

### Data analysis

Clinical and demographic data were summarized using medians and ranges or frequencies and percentages as appropriate. Difference in MIQ_R total score between controls and people with LLA was calculated with Mann–Whitney U Test. The data was not normally distributed and therefore non-parametric analyses were performed.

To address the primary objective and determine the feasibility of measuring brain activation during imagined walking in people with LLA, a group analysis of the fMRI data was performed for the LLA group and healthy control groups using a mixed-effects model via FSL FLAME 1 + 2. One independent third-level analysis was performed to yield the average of LLA participants. For the individual level analyses cluster-based thresholding was performed (*Z* > 3.1, family-wise error (FWE), *p* < 0.05), where the *p*-value was corrected for multiple comparisons.

To address the secondary objectives, three exploratory analyses were performed. The first analysis involved group analysis of the fMRI data performed for the healthy control and LLA groups using a mixed-effects model via FSL FLAME 1 + 2 to explore global activation between the groups. Three independent third-level analyses were performed: (1) average brain activation of control participants; (2) the average brain activation of LLA participants; and (3) the group difference in brain activation between LLA and controls. For the group level analyses, cluster-based thresholding was performed (*Z* > 3.1, family-wise error (FWE), *p* < 0.05), where the *p*-value was corrected for multiple comparisons. The analyses were performed without and with adjustment for age in the group level analyses. Summary table for regions of activation during imagined walking for both groups and comparison between groups using adjusted analysis data were created using MRIcron and Automated anatomical labeling atlas to find the different locations – https://doi.org/10.1016/j.neuroimage.2019.116189.

For the next analysis, the primary motor cortex (M1) was selected as the brain region of interest (ROI) for this study to allow comparison to the existing fMRI literature in lower extremity amputees (Hanspal et al., 2003; Malouin et al., 2009; Haffner et al., 2017). The left M1 ROI, right M1 ROI, and the whole-brain M1 ROI were obtained from the probabilistic Harvard-Oxford cortical structural atlas (as distributed with FSL v6.0) for all participants. The extent of activation in each of the M1 ROIs was quantified for each participant using beta weights and the thresholded z-stat (*Z* > 3.1) image, representing the amount of BOLD signal (%BOLD signal) and volume of activation (VOA) associated with the imaginary task, respectively. A comparison of %BOLD signal and VOA in bilateral primary motor cortices between controls and people with LLA was performed using Mann–Whitney U Test. The effect size was calculated using *r* = z/√n, and the magnitude of the effect size was interpreted as: trivial (<0.20), small (0.20 to <0.50), moderate (0.5 to <0.80) and large (>0.80) (Gregg et al., 2010). A subgroup analysis among the participants with LLA was then performed for the comparison of %BOLD signal and VOA between the ipsilateral and contralateral M1 to the side of amputation using Mann–Whitney U Test. An exploratory analysis compared %BOLD signal and VOA between the M1 ipsilateral and contralateral to the side of amputation stratified by the etiology of amputation using Mann–Whitney U Test.

Finally, to explore the relationship between activation and motor imagery ability, exploratory analysis between %BOLD signal and VOA in the primary motor cortices and motor imagery ability was performed using Spearman ranked correlation bivariate analysis. Statistical analyses of clinical and demographic information, and secondary objectives were performed using SPSS (version 27.0; SPSS, Inc., Chicago, IL, USA) and statistical significance was set at *p* < 0.05.

## Results

We recruited 11 people without LLA as the control group (median age = 28.0 years) and 8 people with LLA (median age = 63.5 years) (Table 1). The motor imagery total score was 84.4 in controls and 89.1 in people with LLA (*p* > 0.05). In participants with LLA, the median M-Plus score was 58.4 and the median Socket Comfort Score was 9.0. All participants with LLA wore their prosthesis on a daily basis and the median duration of prosthesis use was 11.5 years.

**TABLE 1:** Demographic and clinical characteristics of study sample.

	Median [range] or frequency [%]	
	Controls (*n* = 11)	Lower limb amputees (*n* = 8)
Age (years)	28.0 [23–31]	63.5 [35–68]
Number of prescription medications	0 [0–2]	2.0 [0–9]
Gait aid use		
– No gait aid use	11 (100%)	6 (75%)
– Axillary crutches	0	2 (25%)
Motor Imagery Questionnaire		
– Total score	84.4 [61–98]	89.1 [51–98]
– Visual score	43.2 [31–49]	45.5 [27–49]
– Kinesthetic score	41.2 [30–49]	43.6 [24–49]
Fear of falling (n, no)	11 (100%)	8 (100%)
Self-rated health		
– Poor	0	0
– Fair	0	0
– Good	2 (18.2%)	1 (12.5%)
– Very good	7 (63.6%)	5 (62.5%)
– Excellent	2 (18.2%)	2 (25%)
Employment status		
– Employed	8 (72.7%)	4 (50%)
– Unemployed	0	2 (25%)
– Student	3 (27.3%)	0
– Retired	0	2 (25%)
Etiology of amputation		
– Dysvascular		3 (37.5%)
– Trauma		5 (62.5%)
Side of lower limb amputation		
– Right		3 (37.5%)
– Left		5 (62.5%)
Problems with stump: (people could report multiple issues)		
– None		4 (50%)
– Swelling		2 (25%)
– Pain		1 (12.5%)
– Loss of sensation		1 (12.5%)
– Phantom limb pain		1 (12.5%)
Problems with non-amputated limb:		
– None		5 (62.5%)
– Pain		2 (25%)
– Loss of sensation		4 (50%)
Duration using prosthesis (years)		11.5 [1–43]
How many days a week use prosthesis		7 (100%)
Socket comfort score		9.0 [3–10]
Prosthetic limb users survey of mobility		58.4 [48.4–67.1]

### Whole brain fMRI results

**FIGURE 1:**
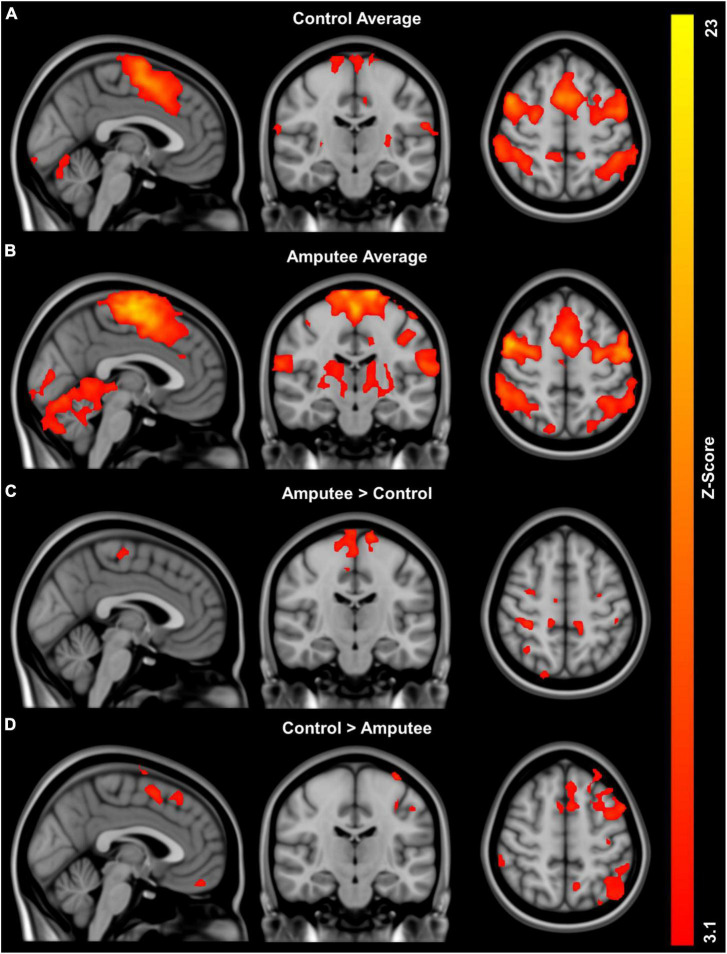
Global activation patterns during imagined walking in people with unilateral transtibial lower limb amputation (*n* = 8) and controls (*n* = 11) using fMRI: **(A)** group average activation controls, **(B)** group average activation for people with lower limb loss, **(C)** regions with greater activity in people with lower limb loss compared to controls adjusted for age, and **(D)** regions with greater activity in controls compared to people with lower limb loss adjusted for age.

Brain areas showing significant positive fMRI responses during imagined walking are identified for controls (Figure 1A) and amputees (Figure 1B). Both groups demonstrate distributed areas of activation bilaterally in multiple areas of the brain, most prominently in the medial surface of the primary motor and somatosensory cortex consistent with representation of the lower limbs. Table 2 provides a quantitative summary of the regions of activation during imagined walking for each group.

**TABLE 2:** Brain regions activated during imagined walking for individual groups and group comparisons between controls and people with unilateral transtibial lower limb amputation.

Group	Brain region	Voxels	MNI coordinates (*x*, *y*, *z*)
Controls	Left middle cingulate gyrus	13544	1.02, 13.6, 34
	Right superior temporal gyrus	3131	52.4, −43.2, 22.1
	Left supramarginal gyrus	2521	−53.4, −46.1, 31.7
	Left crus I of cerebellar hemisphere	511	−37.1, −60.5, −28.3
	Left precuneus	311	−11, −63, 63
	Left lobule VI of cerebellar hemisphere	270	−3, −73.2, −19.1
	Right middle occipital gyrus	259	40.2, −78.9, 33.1
	Left middle occipital gyrus	143	−39.6, −81.4, 30.1
	Left middle cingulate gyrus	116	−12.9, −32.4, 44.2
	Right anterior orbital gyrus	116	24.3, 43.1, −18.8
	Right precuneus	110	9.89, −44.5, 52.9
	Left calcarine	99	−11.6, −103, −5.57
	Right lingual gyrus	57	20.5, −103, −5.57
	Left anterior orbital gyrus	54	−24.3, 43.6, −18,2
Amputees	Left middle cingulate gyrus	10724	0.77, 8.05, 39.2
	Lobule VI of vermis	2412	−0.44, −61.8, −22.5
	Right supramarginal gyrus	2229	54.3, −40.4, 30.2
	Left supramarginal gyrus	2159	−53.8, −43, 31.3
	Right ventral lateral	67	14.1, −10.9, 17.3
	Right pallidum	46	23.8, −8.58, −1.72
	Left caudate	31	−14.6, −5.56, 17.9
	Right superior parietal gyrus	30	18.1, −73.9, 57.8
	Left superior parietal gyrus	26	−19.4, −74.1, 56.3
	Right precuneus	24	10.7, −45.1, 70.9
	Right superior frontal gyrus	20	28.4, 65.1, −8.2
	Left ventral lateral	13	−13.1, −14.9, 9.1
	Left anterior orbital gyrus	13	−24.3, 40.1, −20.3
	Left superior temporal gyrus	13	−69.3, −28.8, 3.68
	Left middle temporal gyrus	9	−53.6, −26.9, −4.1
Control > amputees	Left middle frontal gyrus	2165	−33.4, 20.3, 44.2
	Left angular gyrus	1580	−49.6, −62.4, 26.9
	Right superior temporal gyrus	247	54.2, 12.4, −6.17
	Left postcentral gyrus	224	−35, −25.4, 66.6
	Right temporal middle gyrus	147	61.3, −50, 6.35
	Left inferior frontal gyrus	145	−45.6, 18.7, −0.41
	Left medial orbial gyrus	123	−10.3, 42.8, −22.2
	Right crus I of cerebellar hemisphere	113	39.4, −66.4, −27.6
	Left postcentral gyrus	102	−62.8, −2.9, 23.6
	Right parietal gyrus	83	55.8, −39.5, 51.1
	Left precuneus	64	−9.5, −58.4, 47.2
	Left lateral orbital gyrus	58	−44.6, 50.3, −14.5
Amputees > controls	Left paracentral lobule	810	−8.76, −16.4, 68.6
	Right superior parietal gyrus	559	19.9, −52.9, 67.8
	Left superior parietal gyrus	231	−27.4, −58.1, 66.4
	Right postcentral gyrus	180	34.5, −33.8, 46.3
	Right superior frontal gyrus	131	27.1, −4.32, 70.1
	Right middle frontal gyrus	103	41.2, 51.7, 1.81
	Left inferior parietal gyrus	102	−46.1, −30.8, 44.3
	Right lobule VI of cerebellar hemisphere	96	26.7, −55.7, −21.8
	Left lobule VI of cerebellar hemisphere	92	−26.5, −46.2, −24.9
	Right precentral gyrus	68	30.4, −7.03, 52.6
	Right temporal middle gyrus	61	48.3, −58.2, 0.789
	Right superior parietal gyrus	54	19.4, −80.1, 49.4

Significance level for all clusters listed is *p* < 0.05 corrected for family-wise error; MNI, montreal neurological institute.

Imagined walking reveals significantly greater activation in the people with LLA compared to the controls in the analyses adjusted for age (Figure 1C). Analysis of group differences in fMRI responses revealed multiple distributed areas were significantly more active in controls versus people with in the analyses adjusted for age (Figure 1D). Table 2 provides a quantitative summary of the regions of brain activation during imagined walking in the comparison between groups.

### Primary motor cortex region of interest (ROI) fMRI results

The VOA for the bilateral primary motor cortices was 252.5 voxels for the control group and 822.0 voxels for people with LLA. Mann–Whitney U Test was statistically significant (*U* = 4, z-score = −2.78, *p* = 0.005, effect size = 0.72) for greater VOA for people with LLA. The %BOLD signal for the bilateral primary motor cortices was 0.105 for controls and 0.228 for people with LLA, which was not statistically significant between groups (*U* = 15, z-score = −1.50, *p* = 0.132, effect size = 0.39).

In the people with LLA, the VOA in the ipsilateral M1 to the amputation (467.5 voxels) and in the contralateral M1 to the amputation (410.5 voxels) during imagined walking were not statistically different (*z* = −1.12, *p* > 0.05). Similarly, the %BOLD signal in the ipsilateral M1 to the amputation (0.205) and in the contralateral M1 to the amputation (0.156) during imagined walking were also not statistically different (*z* = −0.700, *p* > 0.05).

In an exploratory analysis among people with LLA stratified by etiology of amputation, there were no statistically significant differences between dysvascular and traumatic etiology groups on: VOA for M1 ipsilateral to the amputation [dysvascular (313.0), trauma (834.0), *z* = −1.64, *p* = 0.143], VOA for M1 contralateral to the amputation [dysvascular (283.0), trauma (642.0), *z* = −1.342, *p* = 0.250], %BOLD for M1 ipsilateral to the amputation [dysvascular (0.098), trauma (0.281), *z* = −1.64, *p* = 0.143], and %BOLD for M 1 contralateral to the amputation [dysvascular (0.1325), trauma (0.167), *z* = −1.64, *p* = 0.143].

### Motor imagery ability

**FIGURE 2:**
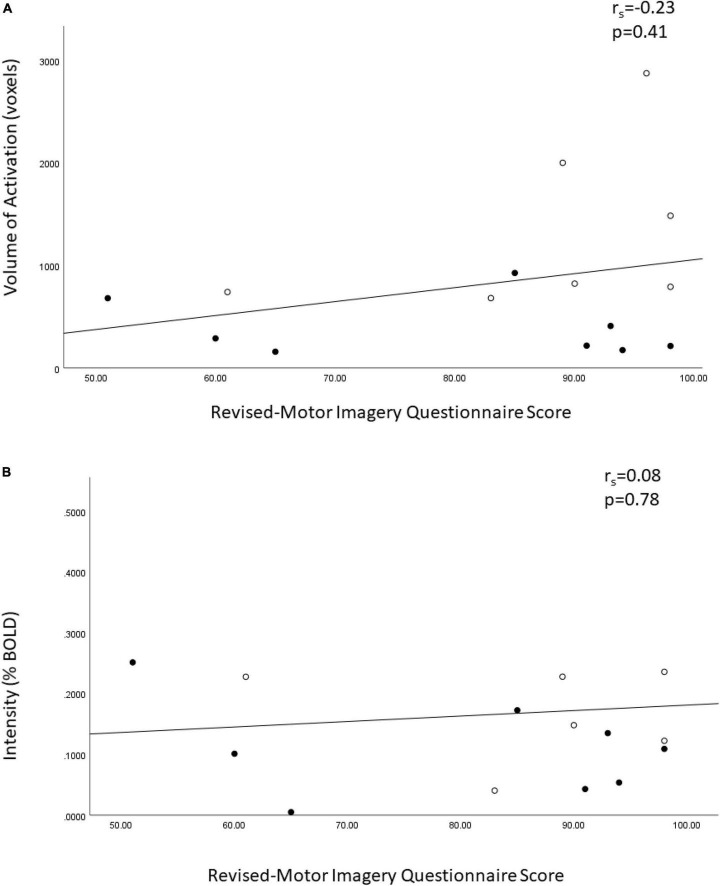
Spearman ranked correlation bivariate analysis of the Revised – Movement Imagery Questionnaire and **(A)** the volume of activation and **(B)** the percent BOLD signal in the bilateral motor cortices in sample of controls and people with unilateral transtibial amputation. o = lower extremity amputees, ● = controls.

Exploratory analysis for the relationship between VOA in the primary motor cortices and the total MIQ-R score demonstrated a small positive correlation (Figure 2A, *r*_s_ = 0.23, *p* = 0.41) (higher motor imagery scores associated with greater volume of activation), which was not statistically significant. The %BOLD had a trivial positive correlation with motor imagery ability and was not statistically significant (Figure 2B).

## Discussion

This study has found that people with LLA demonstrate cortical activation during imagined walking. Additionally, we were able to demonstrate that people with LLA have greater activation in multiple areas of the brain compared to controls during imagined walking. The VOA for the bilateral primary motor cortices was greater for the people with LLA compared to the controls, but there was no difference in the %BOLD signal. There was also no statistically significant difference between VOA and %BOLD signal of the ipsilateral and contralateral M1 to the amputation in people with LLA. Exploratory correlation analysis of motor imagery ability measured using the MIQ-R on the VOA and %BOLD in the bilateral primary motor cortices found no association.

In the current study, imagined walking in the people with LLA demonstrated activation of multiple areas in the brain (e.g., primary motor cortex, occipital cortex, cerebellum, prefrontal cortex). The distributed pattern of brain activation during imagined walking in the people with LLA is consistent with patterns reported in the systematic review by Hamacher et al. (2015) among healthy and patient populations during imagined walking. The systematic review by Hamacher et al. (2015) did not include any studies evaluating people with LLA, so direct comparison across studies on people with LLA is not possible. Wang et al. (2021) found sensorimotor cortex organization is preserved in people with LLA, our study also demonstrated increased activation on the medial surface of the primary motor and sensory cortices that corresponds to lower limb topographical representation. Alterations in the primary motor homunculus of the amputated lower limb is another area for further exploration for imagined walking. Additionally, evaluation of activation patterns over different complexity of imagined walking tasks (walking forward, backward, dual-task testing) and the relationship of activation patterns in imagined walking to real-world tasks of walking.

Multiple factors have been identified that can impact brain activation levels during imagined walking that are related to age, complexity of the imagined walking task and having a lower limb amputation. Simoes et al. (2012) found expansion of activation maps of the amputated lower limb for both primary sensory and motor areas into neighboring regions as a consequence of functional plasticity after the amputation. Additionally, the sample of people with LLA in our study was older than the controls. Research by Allali et al. (2014) found healthy young and older adults demonstrated an overlap of brain regions recruited during imagined walking that included bilateral primary motor cortex, supplementary motor cortex, prefrontal cortex and cerebellum. The brain regions recruited are consistent with our results for people with LLA. Allali et al. (2014) also found that healthy older adults demonstrated greater activation in the prefrontal cortex and supplemental motor cortex compared to healthy young adults during imagined walking.

Though, Zwergal et al. (2012) found the locomotor network that includes the prefrontal cortex, basal ganglia, brainstem and cerebellar locomotor centers are preserved in healthy older adults with imagined walking. Another feature that could be relevant in the level of cortical activation is the complexity of the imagined walking tasks, such as walking backward, are associated with higher activation patterns in the prefrontal cortex for older adults compared to younger adults (Hamacher et al., 2015). Ambulation with a prosthesis is a complex motor task and people with LLA using a prosthesis report having to think about every step they take which indicates ongoing conscious higher-order cognitive processing for the learned activity (Miller et al., 2001). Our sample of people with LLA were high functioning community ambulators using their prosthesis on a daily basis and had many years of experience using their prosthesis. Yet, our sample of people with LLA had greater VOA compared to the controls. The increased activation observed in the current study among the people with LLA may reflect the difference in task difficulty on imagined walking between the two groups.

Among people with lower limb amputations, the role of the amputation etiology may be relevant to brain activation patterns. Functional magnetic resonance imaging (fMRI) is the optimal method to evaluate task-based brain activation patterns as it can infer changes in neuronal activation using blood oxygen level dependent (BOLD) contrast, which incorporates changes in regional blood flow, blood volume and levels of deoxyhemoglobin induced by neuronal activation. While there are multiple etiologies that can result in lower limb amputation, 80% of LLA in Canada result from dysvascular disease (Lamb et al., 2005). Future research should evaluate brain activation across amputation etiologies as they may yield different activation patterns due to vascular changes present in dysvascular disease. Additionally, it is unknown whether these patterns of activation correlate with mobility performance in people with LLA of either etiology.

Lastly, an exploratory analysis of the relationship of motor imagery ability and brain activation was evaluated in the study as well. The Revised – Movement Imagery Questionnaire (MIQ-R) assesses motor imagery ability from the first-person perspective (Gregg et al., 2010). Our exploratory analysis did not find a statistically significant relationship between the MIQ-R score and the VOA or %BOLD signal in the primary motor cortices among the whole sample. Motor imagery ability in known to vary between individuals representing a continuum of vividness and is not an all-or-none phenomena. Ability is influenced by the nature of any brain injury (e.g., stroke) (Malouin and Richards, 2010), premorbid ability (Malouin and Richards, 2010) and exposure to motor imagery practice as demonstrated in athletes and musicians (Ladda et al., 2021). After limb amputation, mental representation of motor action for the amputated limb is retained but is more difficult for people to access and is modulated by motor imagery practice (Malouin et al., 2009). Importantly, the finding that people with LLA ambulating with a prosthesis report having to think about every step they take could be considered mental practice of walking and may facilitate motor imagery ability (Miller et al., 2001). In the plot of MIQ-RS score versus VOA, high scores and high volumes of activation were achieved by people with LLA and controls. The MIQ-R is one of best-evaluated assessment tools of motor imagery ability and has been found to demonstrate sufficient psychometric properties for the evaluation of motor imagery ability (Suica et al., 2022). It still needs to be determined if this is the most appropriate tool to use for people with LLA due to the limited tasks for the lower extremity.

The literature regarding the role of motor imagery to facilitate gait re-education for people with LLA learning to use a prosthesis is limited. A case study of a person with LLA demonstrated improved gait after a mental imagery training program that focused on functional tasks of walking, balancing and reaching (Matalon et al., 2021). A small randomized controlled trial evaluated the effect of motor imagery training of gait in combination with real execution of gait retraining compared to a control group who performed only non-motor imagery tasks (Cunha et al., 2017). The authors reported the group with real gait training and motor imagery training improved gait performance over the control group, though a definitive conclusion of the merits of the addition of motor imagery training to usual gait training protocols cannot be made (Cunha et al., 2017). Unlike the work by Cruz et al. (2003) that was unable to detect cortical activation with motor imagery of knee flexion/extension, we have established a protocol that can be used to evaluate motor imagery training programs in people with unilateral transtibial amputations of mixed etiology.

There are several limitations to this study that should be considered. As a pilot study, the intent was to determine if cortical activation was possible to elicit in people with LLA while performing imagined walking. Our study was able to successfully demonstrate cortical activation in people with LLA, but the secondary analyses were exploratory and require further confirmation with an adequately powered study. Additionally, recruitment for this study was a challenge to find people with LLA who were interested, met the criteria for having an MRI and willing to come for a two-visit research protocol. Most importantly, there was an age difference between the groups which may have contributed to the observed difference in activation patterns in people with LLA compared to younger controls. To address this issue, the analyses comparing the groups was adjusted for age. To better disentangle age, consequences of cortical plasticity after amputation, and the complexity of the imagined gait task participants with LLA should be matched to controls on age. Our study also attempted to control for phantom limb pain which has been found to impact cortical reorganization post-amputation and brain activation patterns (Gunduz et al., 2020).

## Conclusion

This study has demonstrated that people with LLA demonstrate cortical activation during imagined walking. The volume of activation for the bilateral primary motor cortices was greater for the people with LLA compared to a group of younger controls. Exploratory correlation analysis of motor imagery ability on the volume of activation and intensity of activation in the bilateral primary motor cortices found no relationships. The protocol developed in this pilot study provides the foundation to evaluate the effects of a gait training program that incorporates mental imagery in conjunction with usual rehabilitation practices compared to usual care alone.

## Data availability statement

The raw data supporting the conclusions of this article will be made available by the authors, without undue reservation.

## Ethics statement

The studies involving human participants were reviewed and approved by the Health Sciences Research Ethics Board at Western University, London, Ontario, Canada. The patients/participants provided their written informed consent to participate in this study.

## Author contributions

SH: concept and design of the study, acquisition of subject’s data, interpretation of data and statistical analysis, writing first draft, and writing final version of manuscript. AM: acquisition of subject’s data, writing first draft, and writing final version of manuscript. AC: concept and design of the study, interpretation of data and statistical analysis, writing first draft, and writing final version of manuscript. RB: concept and design of the study, acquisition of subject’s data, interpretation of data and statistical analysis, and writing final version of manuscript. RV and MP: concept and design of the study, acquisition of subject’s data, writing first draft, and writing final version of manuscript. All authors contributed to the article and approved the submitted version.
